# A conserved arginine/lysine-based motif promotes ER export of KCNE1 and KCNE2 to regulate KCNQ1 channel activity

**DOI:** 10.1080/19336950.2019.1685626

**Published:** 2019-11-03

**Authors:** Bin Hu, Wen-Ping Zeng, Xia Li, Umar Al-Sheikh, San-You Chen, Jiuping Ding

**Affiliations:** a Key Laboratory of Molecular Biophysics of the Ministry of Education, College of Life Science and Technology, Huazhong University of Science and Technology, Wuhan, Hubei, China; b School of Life Sciences, University of Science and Technology of China, Hefei, Anhui, China; c CAS Key Laboratory of Microscale Magnetic Resonance and Department of Modern Physics, University of Science and Technology of China, Hefei, Anhui, China

**Keywords:** KCNQ1, KCNE1, KCNE2, forward trafficking, ER export signal

## Abstract

KCNE β-subunits play critical roles in modulating cardiac voltage-gated potassium channels. Among them, KCNE1 associates with KCNQ1 channel to confer a slow-activated *I*_Ks_ current, while KCNE2 functions as a dominant negative modulator to suppress the current amplitude of KCNQ1. Any anomaly in these channels will lead to serious myocardial diseases, such as the long QT syndrome (LQTS). Trafficking defects of KCNE1 have been reported to account for the pathogenesis of LQT5. However, the molecular mechanisms underlying KCNE forward trafficking remain elusive. Here, we describe an arginine/lysine-based motif ([R/K](S)[R/K][R/K]) in the proximal C-terminus regulating the endoplasmic reticulum (ER) export of KCNE1 and KCNE2 in HEK293 cells. Notably, this motif is highly conserved in the KCNE family. Our results indicate that the forward trafficking of KCNE2 controlled by the motif (KSKR) is essential for suppressing the cell surface expression and current amplitude of KCNQ1. Unlike KCNE2, the motif (RSKK) in KCNE1 plays important roles in modulating the gating of KCNQ1 in addition to mediating the ER export of KCNE1. Furthermore, truncations of the C-terminus did not reduce the apparent affinity of KCNE2 for KCNQ1, demonstrating that the rigid C-terminus of KCNE2 may not physically interact with KCNQ1. In contrast, the KCNE1 C-terminus is critical for its interaction with KCNQ1. These results contribute to the understanding of the mechanisms of KCNE1 and KCNE2 membrane targeting and how they coassemble with KCNQ1 to regulate the channels activity.

## Introduction

Voltage‐gated K^+^ (K_v_) channels play important roles in regulating cellular excitability by controlling the membrane potential. The KCNE family (KCNE1–KCNE5) functions as auxiliary β-subunit to modulate the subcellular localization, ion selectivity, gating and pharmacology of K_v_ channels in muscles and epithelia [1]. KCNQ1 (Kv7.1 or KvLQT1), is a K_v_ channel α-subunit with six transmembrane domains (S1–S6) [2,3], can associate with all the five KCNE β-subunits, resulting in different biophysical properties. In cardiac cells, KCNQ1 interacts with KCNE1 to generate a slow-activated and enhanced current known as *I*_Ks_, which is essential for proper repolarization of the cardiac action potential [4,5]. KCNE2 reduces the current amplitude of KCNQ1 to form a background-like current [6]. In addition, KCNE2 can also suppress the current amplitude of KCNQ1/KCNE1 complex [7,8]. KCNE3 coassembles with KCNQ1 to confer constitutive activity [9,10]. KCNE4 and KCNE5 inhibit the channel activity of KCNQ1 [11,12]. Any anomaly in these channels will lead to serious myocardial diseases, like the long QT syndrome (LQTS). LQTS is a disease characterized by delayed cardiac repolarization and prolonged QT interval on the electrocardiogram (ECG), resulting in serious arrhythmias, ventricular fibrillation and cardiac arrest [13]. Approximately 75% of patients with LQTS carry mutations in 5 genes: *KCNQ1* (LQT1), *KCNH2* (LQT2), *SCN5A* (LQT3), *KCNE1* (LQT5), and *KCNE2* (LQT6) [14].

Trafficking of transmembrane proteins such as ion channels is complex [15]. Protein transport between the ER and Golgi is a key early event in forward trafficking and involves specific amino acid motifs so called ER export signals [16]. For instance, many ER export signals have been identified at cytoplasmic tail of membrane proteins. The most common ER export signals can be divided into three classes: diacidic, dihydrophobic and dibasic, e.g. D/E-X-D/E [17,18], FCYENE [19], FXXXFXXXF [20], VXXSL [21], YMVIEE [22], [R/K](X)[R/K] [23]. In most cases, cargo proteins containing the diacidic or dihydrophobic signals can associate with the coat protein complex II (COPII) component Sec24 for further trafficking [24–26]. However, in few cargo proteins such as glycosyltransferases, the dibasic motif, [R/K](X)[R/K], functions as an ER export signal through its interactions with Sar1 [23,27,28], another component of the COPII.

Protein processing and trafficking are of great importance in controlling channel characteristics. Trafficking defects of the channel proteins are related to the pathogenesis of LQTS [29]. For example, most missense mutations in *KCNH2* give rise to defects in protein assembly and cell surface trafficking [30]. It has been reported that abnormal trafficking of KCNE1 accounts for the occurrence of LQT5 [31–33]. Therefore, unraveling the subcellular localization of channel proteins and their assembly process is of great significance for understanding the pathogenesis of LQTS and other ion channel diseases. However, little is known about the trafficking determinants of the auxiliary KCNE β-subunits.

In this study, we aimed to characterize the molecular determinants accounting for KCNE1 and KCNE2 forward trafficking. We identified an arginine/lysine-based motif, [R/K](S)[R/K][R/K], in the proximal C-terminus of KCNE1 and KCNE2 that is essential for efficient ER export and regulation of KCNQ1 functions. This motif is highly conserved in the KCNE family. Besides, co-immunoprecipitation assays indicated that the KCNE2 C-terminus may not physically interact with KCNQ1, while the KCNE1 C-terminus is important for its interaction with KCNQ1. Since many mutations in the C-terminus of KCNE1 and KCNE2 have been reported to result in LQTS [34], comprehending the roles of KCNE1 and KCNE2 C-terminus in controlling their trafficking and modulating channel functions is of great importance.

## Experimental procedures

### Constructs and mutations

**Figure 1.**
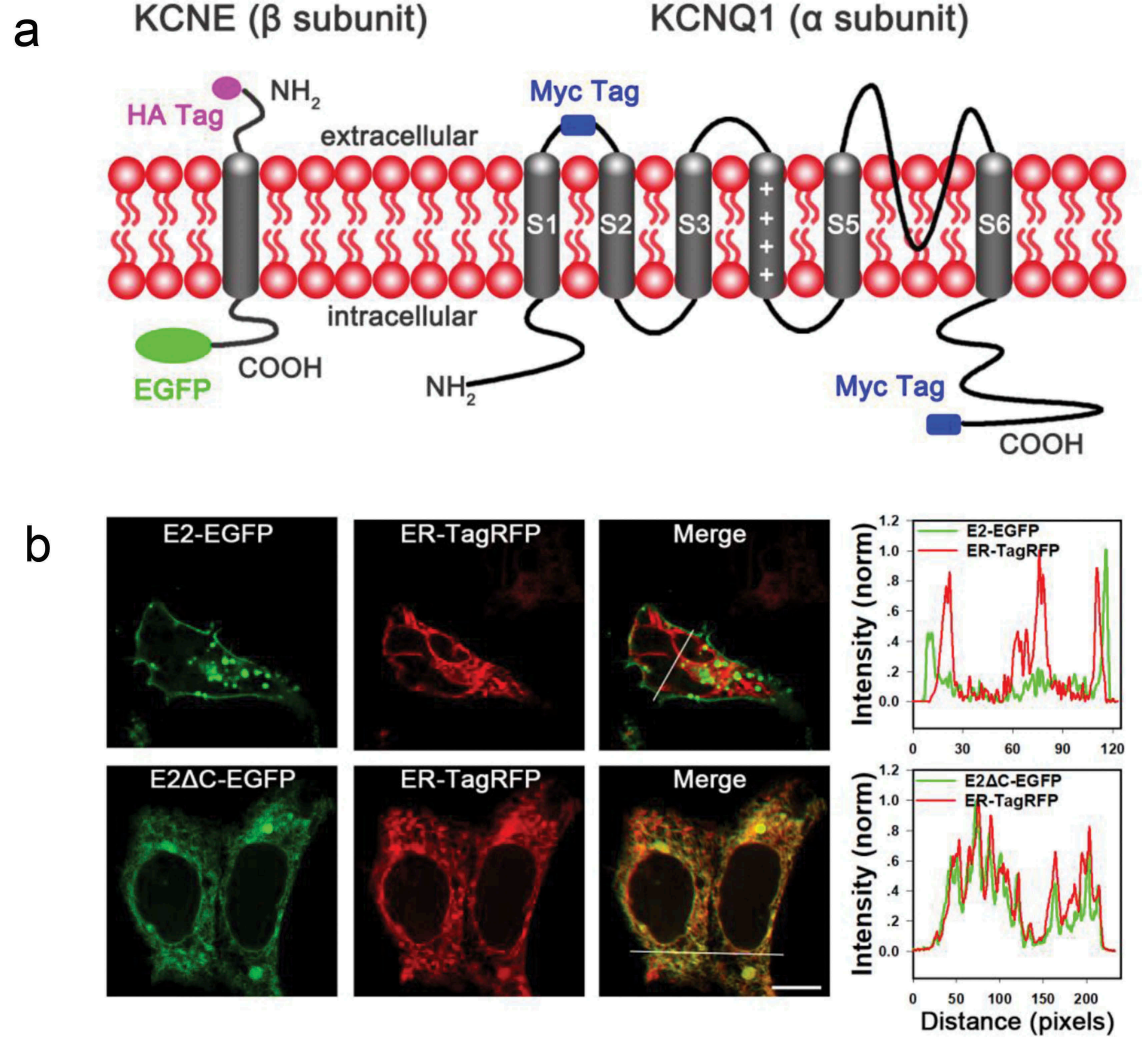
The C-terminus of KCNE2 regulates ER export of the protein to the plasma membrane in HEK293 cells. (a) Topology diagrams of modified KCNQ1 (Q1) and KCNE (KCNE2 (E2) or KCNE1 (E1)) subunits. KCNQ1 were tagged with a Myc-epitope in the middle of S1–S2 linker or at its C-terminus. KCNE2 or KCNE1 was fused with an EGFP to its C-terminus or tagged with a HA-epitope at its N-terminus. (b) Confocal images of HEK293 cells co-transfected with indicated E2* (WT and mutant E2)-EGFP and ER-TagRFP. The merged images show the combination. The scale bar is 10 μm. The right column shows the pixel intensity profiles of crossed sections indicated by the white line.

For the constructs KCNE2(E2)-EGFP and KCNE1(E1)-EGFP, the EGFP cDNA was amplified by PCR using Pfu polymerase (Fermentas, Biotech) and cloned into pcDNA3.1 (+), then KCNE2 or KCNE1 cDNA lacking stop codon were amplified and fused in frame to the N-terminus of EGFP. Using the same method, the truncations of KCNE2 or KCNE1 were made by deleting appropriate amino acids, fused to the N-terminus of EGFP and cloned into pcDNA3.1 (+). The construct Myc-KCNQ1 (Q1) was prepared as previously described [35]. Q1-Myc was prepared by adding a Myc tag to the C-terminus of KCNQ1 by PCR and cloned into pcDNA3.1 (+) (Figure 1a). HA-E2 and HA-E1 were made by introducing a HA tag to the N-terminus of KCNE2 or KCNE1 by PCR and cloned into pcDNA3.1 (+). The ER marker (ER-TagRFP), which was a gift from Dr. Rongying Zhang (Huazhong University of Science and Technology), was constructed by adding the human calreticulin signal sequence (MLLSVPLLLGLLGLAVA) to the N-terminus of TagRFP and cloned into pcDNA3.1 (+). All constructs and mutations were verified through direct DNA sequencing.

### Cell culture and transient transfection

HEK293 cells were cultured and transfected as previously described [36]. For immunofluorescence imaging, the plasmid pcDNA3.1-TdimerII was introduced to identify transfected cells. For co-transfection experiments, the ratio of KCNQ1 coding plasmid to KCNE2 or KCNE1 coding plasmid was 1:1.

### Immunofluorescence

After transfection for 22–24 h, HEK293 cells were washed and fixed with 2% paraformaldehyde (PFA) in PBS for 12 min, followed by 5 min × 3 washes with PBS. Then cells were blocked with 2% BSA in PBS for 1 h and incubated with mouse monoclonal anti-HA antibody (Millipore, 1:300) or mouse monoclonal anti-Myc antibody (Abcam, 1:300) for 3 h. Cells were washed with PBS 5 times and 1% BSA once. After incubation with Alexa Fluor 488-conjugated goat anti-mouse IgG (H + L) (Invitrogen, 1:300) for 1–1.5 h, the cells were washed with PBS for 6 times. All the procedures were performed at room temperature (22–25°C).

Fluorescence images were visualized by using a spinning-disk confocal imaging system (CSU-X1 Nipkow Yokogawa, Japan) equipped with an Olympus IX-71 inverted microscope (Olympus Corp., Japan). 16-bit digital images were obtained with oil-immersion objective (100 ×, NA 1.30) and an EM CCD camera (DU897K, ANDOR iXon, United Kingdom).

### Electrophysiology

The day after transfection, whole-cell patch clamp recordings were carried out in HEK293 cells in the bath solution (in mM: 150 NaCl, 4 KCl, 2 CaCl_2_, 1 MgCl_2_, 10 HEPES (pH 7.0)) at room temperature (22–25°C). Patch pipettes were pulled from borosilicate glass capillaries with resistance of 2–3 megohms and filled with the pipette solution (in mM: 5 EGTA, 140 KCl, 10 NaCl, 1 MgCl_2_, 10 HEPES (pH 7.0)). The recordings were carried out using a Multiclamp 700B amplifier with a Digidata 1440A A/D converter and pClamp software (Axon Instruments). The currents were typically digitized at 20 kHz and filtered at 5 kHz. All the chemicals were purchased from Sigma-Aldrich. When recording *I*_Ks_, an *I*_Ks_ selective blocker (HMR 1556; MedChemExpress) was applied to test the non-*I*_Ks_ current. Briefly, whole-cell recording was performed on Q1/E1-expressing HEK293 cells first, then HMR 1556 was added to the bath solution at a final concentration of 1 μM and incubated for 5 min before the non-*I*_Ks_ current was tested.

### Data analysis

The quantification of the fluorescence intensities of Myc-Q1, HA-E2 or HA-E1 was performed with ImageJ 1.45 (Wayne Rasband, National Institutes of Health). Electrophysiology data were analyzed with Clampfit 10.3 (Axon Instruments, Inc.) and SigmaPlot 14.0 (SPSS, Inc.) software. The conductance-voltage (G-V) relationship were fitted by the Boltzmann equation: G/G_max_ = (1 + exp((V-V_50_)/κ))^−1^, where V_50_ is the voltage at which the conductance (G) is half the maximum conductance (G_max_) and κ is a factor affecting the steepness of the activation. For *I*_Ks_ analysis, the non-*I*_Ks_ current was subtracted from the recorded *I*_Ks_ current using an offline subtraction strategy. Unless stated otherwise, the data were presented as mean ± SEM. Student’s t-test was used to evaluate the statistical significance. P < 0.05 was considered significant (* p < 0.05, ** p < 0.01, *** p < 0.001).

### Co-immunoprecipitation

After transfection for 36–48 h, HEK293 cells were lysed in 700 μL lysis buffer (25 mM Tris-HCl pH 7.4, 150 mM NaCl, 1% Nonidet P-40, 5 mM EDTA, 1 mM PMSF, with complete mini EDTA-free protease inhibitor cocktail tablets(Roche)) for 15 min and then scraped into 1.5 mL tube, incubated for 30 min with gentle agitation at 4°C. Insoluble material was removed by centrifugation (14,000 rpm) at 4°C for 20 minutes. Supernatants were collected and pre-cleared with dynabeads protein G (Novex by Life Technologies), 40 μL sample was used for input and the rest for Co-IP. Extracted proteins were incubated with 2 μg of mouse monoclonal anti-HA tag (Millipore) at 4°C overnight with gentle agitation on a platform shaker, followed by incubation with dynabeads protein G for 3 h. The beads were washed with lysis buffer 3 times to remove nonspecific binding. Immunoprecipitates were prepared in 40 μL of 2 x SDS-PAGE loading buffer (2% SDS (w/v), 20% glycerol (v/v), 2% β-mercaptoethanol (v/v), 0.2% bromphenol blue (w/v), and 100 mM Tris-HCl (pH 6.8)) for 30 min at room temperature and resolved on a 10% or 15% SDS-PAGE gel. Immunoprecipitations with mouse normal IgG (IgG-IP) were used as negative controls.

### Western blotting

For immunoblotting, immunoprecipitates were transferred to nitrocellulose membrane. Membranes were incubated with 5% milk in 1× TBS-0.05% Tween-20 for 2 h at room temperature, and then incubated with HRP-conjugated Mouse anti-HA antibody (Sigma, 1:4000) or rabbit polyclonal anti-Myc antibody (Sigma, 1:10,000) in 1× TBS-0.05% Tween-20. The membranes were subsequently washed four times for 10 minutes each with 1× TBS-0.05% Tween-20. Immunoreactivity was visualized by using the ECL detection system directly or after incubation with the peroxidase-conjugated goat anti-rabbit IgG antibody (Millipore, 1:10,000) for 2 h.

## Results

### The C-terminus of KCNE2 (E2) is critical for its ER export in HEK293 cells

All the five KCNE β-subunits harbor one transmembrane (TM) segment, an extracellular N-terminus and an intracellular C-terminus (Figure 1a) [37,38]. Wild-type (WT) KCNE2 presented clear membrane targeting in HEK293 cells (Figure 1b). However, we found that the C-terminal (amino acid residues 73–123) deletion mutant E2ΔC showed strong intracellular retention. To further determine its subcellular localization, we examined HEK293 cells, which were transiently co-transfected with ER marker (ER-TagRFP, see *Experimental Procedures*) and E2-EGFP or E2ΔC-EGFP. The results showed that E2ΔC-EGFP clearly colocalized with the ER marker, as shown in the merged image and the pixel intensity profile of crossed section indicated by the white line (Figure 1b). Thus, we speculated that the C-terminus was a major determinant in promoting the ER export of KCNE2.

### A specific sequence in the proximal C-terminus is essential for the ER export of KCNE2

**Figure 2.**
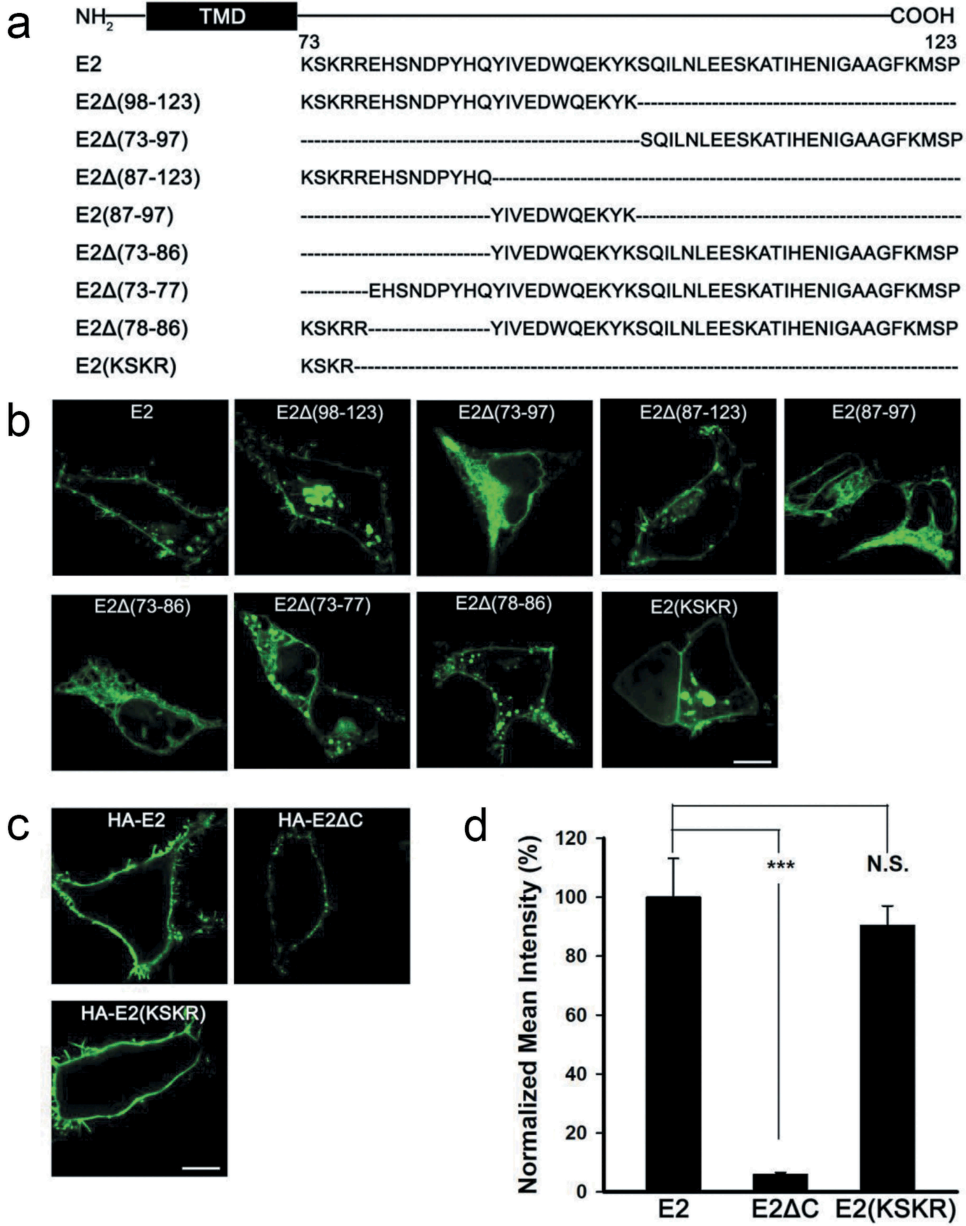
A specific C-terminal sequence proximal to the transmembrane domain of KCNE2 is sufficient for ER export. (a) C-terminal truncations of KCNE2 are displayed, where the dashed lines represent the deleted amino acids. (b) Subcellular localization of the KCNE2 truncations that were expressed as EGFP fusion proteins in HEK293 cells. (c) Immunofluorescence images of HEK293 cells transfected with HA-E2* as indicated. The cells were immunostained with mouse anti-HA primary antibody and goat anti mouse Alexa Fluor 488-conjugated second antibody under nonpermeabilized conditions. The scale bar is 10 μm. (d) Statistics of the relative surface fluorescence intensities of HA-E2* were plotted as indicated. n = 70, 80, 80 for E2, E2ΔC, E2(KSKR). *** p < 0.001, N.S. represents no significance (Student’s t-test).

Protein transport is often governed by molecular recognition of trafficking signals at different stages of the secretory pathway. To explore whether a forward trafficking signal resides in the C-terminus of KCNE2, we made a series of C-terminal truncations of KCNE2 (Figure 2a) that were fused with EGFP, and then examined their subcellular localization in HEK293 cells. As shown in Figure 2b, truncations lacking the proximal C-terminal amino acids, E2Δ(73–97), E2(87–97), E2Δ(73–86) and E2Δ(73–77), abolished the plasma membrane fluorescence of KCNE2. In contrast, truncations containing the KSKRR sequence in the proximal C-terminus, E2Δ(98–123), E2Δ(87–123) and E2Δ(78–86), displayed significant cell surface expression (Figure 2b). These results implied the importance of the KSKRR sequence in determining the surface expression of KCNE2. Since the KSKR sequence is highly conserved in KCNE1–KCNE5 (Figure S1B) [37], we made another truncation E2(KSKR) containing only the KSKR sequence in the C-terminus (Figure 2a) to examine whether it is sufficient for promoting the ER export of KCNE2. To our expectation, E2(KSKR) showed obvious cell surface expression (Figure 2b).

Considering that the use of fusion fluorescent protein EGFP might influence the trafficking of KCNE2 subunits, we inserted a small tag, HA tag, at the extracellular N-terminus of KCNE2 and its mutants (Figure 1a) to visualize their cell surface expression by immunofluorescence with nonpermeabilized cells. As shown in Figure 2c, HA-E2 and HA-E2(KSKR) exhibited robust surface fluorescence; in contrast, HA-E2ΔC showed a very low surface expression level. Quantification of the surface fluorescence intensities indicated that the plasma membrane expression level of HA-E2(KSKR) (90.4 ± 6.7%) was similar to that of HA-E2 (100.0 ± 13.2%), while the surface fluorescence intensity of HA-E2ΔC was only 6% of HA-E2 (Figure 2d). These results are consistent with the EGFP-labeling data, confirming that the KSKR sequence is essential for promoting the ER export of KCNE2 to the cell surface.

### ER export of KCNE2 is dependent on the positively charged residues in the proximal C-terminus

**Figure 3.**
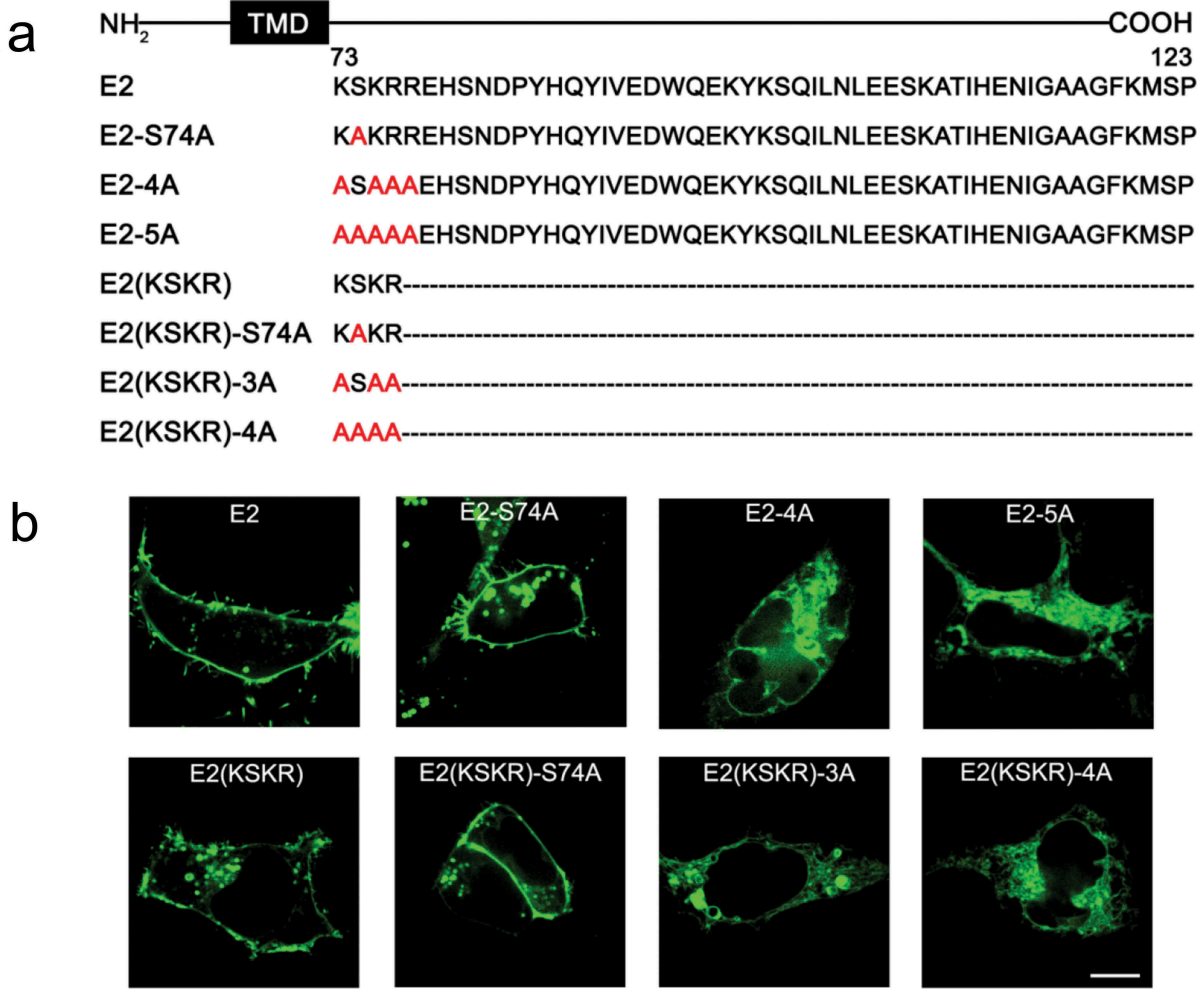
ER export of KCNE2 is dependent on the positively charged residues in the proximal C-terminus. (a) C-terminal mutations of KCNE2 are displayed, where the dashed lines represent the deleted amino acids and the red represents the amino acids mutated to alanine. (b) Confocal images of the KCNE2 mutations that were expressed as EGFP fusion proteins in HEK293 cells.

The phosphorylation state of membrane proteins has been reported to determine their export from ER [39,40]. Since the amino acid serine (S) in the KSKR sequence is fully conserved among all the five KCNE β-subunits (Figure S1B) and is a putative PKC phosphorylation site [41], we examined the potential role of this residue in determining the ER export of KCNE2. We mutated the serine to alanine (A) in WT KCNE2 and E2(KSKR) (Figure 3a). Surprisingly, both E2-S74A and E2(KSKR)-S74A exhibited robust surface fluorescence (Figure 3b), indicating that the serine residue is not likely to control the ER export of KCNE2.

Positively charged amino acids have also been reported to facilitate ER export [42,43]. Thus the positively charged amino acids (K and R) in KSKRR or KSKR may be responsible for the forward trafficking of KCNE2. As expected, when we mutated these residues to alanine (Figure 3a), E2-4A and E2(KSKR)-3A showed dramatic intracellular retention, and similar results were observed in E2-5A and E2(KSKR)-4A (Figure 3b). From these data, we conclude that the positively charged residues in the proximal C-terminus are engaged in controlling the ER export of KCNE2.

### Forward trafficking of KCNE2 is essential for the regulation of KCNQ1 activity

**Figure 4.**
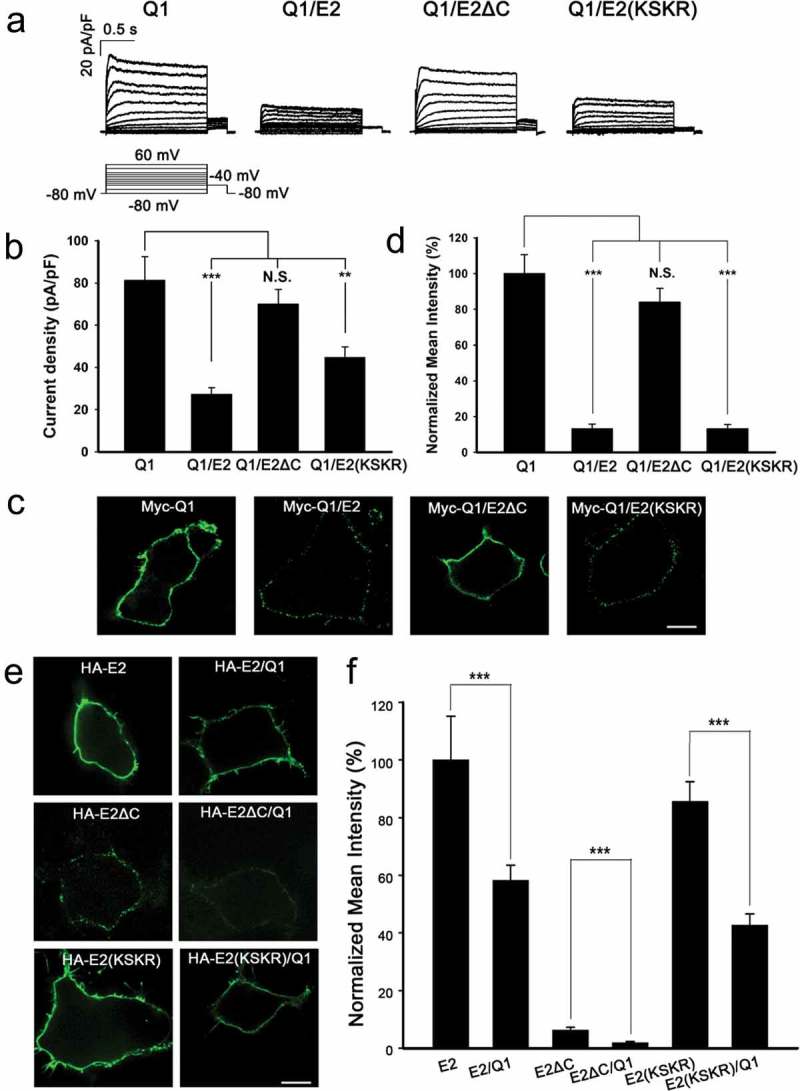
Forward trafficking of KCNE2 is essential for the regulation of KCNQ1 activity. (a) Current traces of Q1 and Q1/E2* expressed in HEK293 cells. The voltage protocol was placed at the bottom. All the currents were shown as current density in pA/pF. (b) Statistics of the current densities at 60 mV as indicated. n = 15, 15, 15, 14 for Q1, Q1/E2, Q1/E2ΔC, Q1/E2(KSKR). (c) Immunofluorescence images of HEK293 cells transfected with Myc-Q1 or Myc-Q1/E2*. The cells were immunostained with mouse anti-Myc primary antibody and goat anti mouse Alexa Fluor 488-conjugated second antibody under nonpermeabilized conditions. The scale bar is 10 μm. (d) Statistics of the relative surface fluorescence intensities of Myc-Q1 were plotted as indicated. n = 62, 61, 76, 68 for Q1, Q1/E2, Q1/E2ΔC, Q1/E2(KSKR). *** p < 0.001, ** p < 0.01, N.S. represents no significance (Student’s t-test). (e) Immunofluorescence images of HEK293 cells transfected with HA-E2* or HA-E2*/Q1. The cells were detected with mouse anti-HA antibody and goat anti mouse Alexa Fluor 488-conjugated second antibody under nonpermeabilized conditions. The scale bar is 10 μm. (f) Normalized fluorescence intensities of HA-E2* for the transfections of HA-E2* or HA-E2*/Q1. n = 72, 80, 82, 83, 84, 82 for E2, E2/Q1, E2ΔC, E2ΔC/Q1, E2(KSKR), E2(KSKR)/Q1. *** p < 0.001 (Student’s t-test).

KCNE2 can interact with KCNQ1 and decrease KCNQ1 current amplitude [6]. Our previous study demonstrated that KCNE2 attenuated KCNQ1 current amplitude by reducing the surface expression of KCNQ1 [36].To investigate the role of KCNE2 forward trafficking in regulating the function of KCNQ1, we performed whole-cell patch clamp recordings in HEK293 cells transfected with Q1 alone or co-transfected with Q1/E2, Q1/E2ΔC or Q1/E2(KSKR), respectively (Figure 4a). Consistent with previous study [6], the current density of Q1/E2 (27.3 ± 2.9 pA/pF) decreased remarkably compared to Q1 (81.2 ± 11.3 pA/pF). Surprisingly, we found that the current density of Q1/E2ΔC (69.9 ± 7.0 pA/pF) was similar to that of Q1 alone, while the current density of Q1/E2(KSKR) (44.7 ± 5.0 pA/pF) decreased obviously (Figure 4b). To verify whether these changes were caused by the effects on KCNQ1 surface expression by E2* (WT and mutant KCNE2), we also quantitatively measured the surface fluorescence intensity of KCNQ1 by inserting a Myc-epitope into the S1–S2 linker of KCNQ1 (named Myc-Q1, Figure 1a). As expected, the surface fluorescence intensity of Myc-Q1/E2 (13.2 ± 2.5%) or Myc-Q1/E2(KSKR) (13.2 ± 2.4%) was significantly lower than that of Myc-Q1 alone (100.0 ± 10.7%); On the contrary, the surface fluorescence intensity of Myc-Q1/E2ΔC (83.9 ± 7.8%) was similar to that of Myc-Q1 (Figure 4c,d). These results suggested that the KSKR sequence is essential for suppressing the surface expression and current amplitude of KCNQ1 channels, probably by controlling the forward trafficking of KCNE2.

Roura-Ferrer et al. found that the membrane targeting of KCNE2 was also impaired when co-expressed with KCNQ1 [44]. Similarly, our results showed that the surface expression of HA-E2 or HA-E2(KSKR) was significantly reduced by the presence of KCNQ1 (HA-E2 vs HA-E2/Q1: 100.0 ± 15.2% vs 58.2 ± 5.4%; HA-E2(KSKR) vs HA-E2(KSKR)/Q1: 85.6 ± 6.9% vs 42.6 ± 3.9%) (Figure 4e,f). Interestingly, the surface expression of HA-E2ΔC also decreased when co-expressed with KCNQ1 (HA-E2ΔC vs HA-E2ΔC/Q1: 6.3 ± 0.9% vs 1.9 ± 0.4%) (Figure 4e,f), which might explain why Q1/E2ΔC showed slight decrease both in current density and surface expression (Figure 4b,d), and maybe for a reason that a small amount of E2ΔC can escape from the ER to suppress the surface expression of KCNQ1.

Based on these results, we conclude that the KSKR sequence determining the ER export of KCNE2 is essential for prevention of KCNQ1 membrane targeting to suppress KCNQ1 activity.

### The conserved RSKK sequence in the proximal C-terminus of KCNE1 is required for its efficient ER export

**Figure 5.**
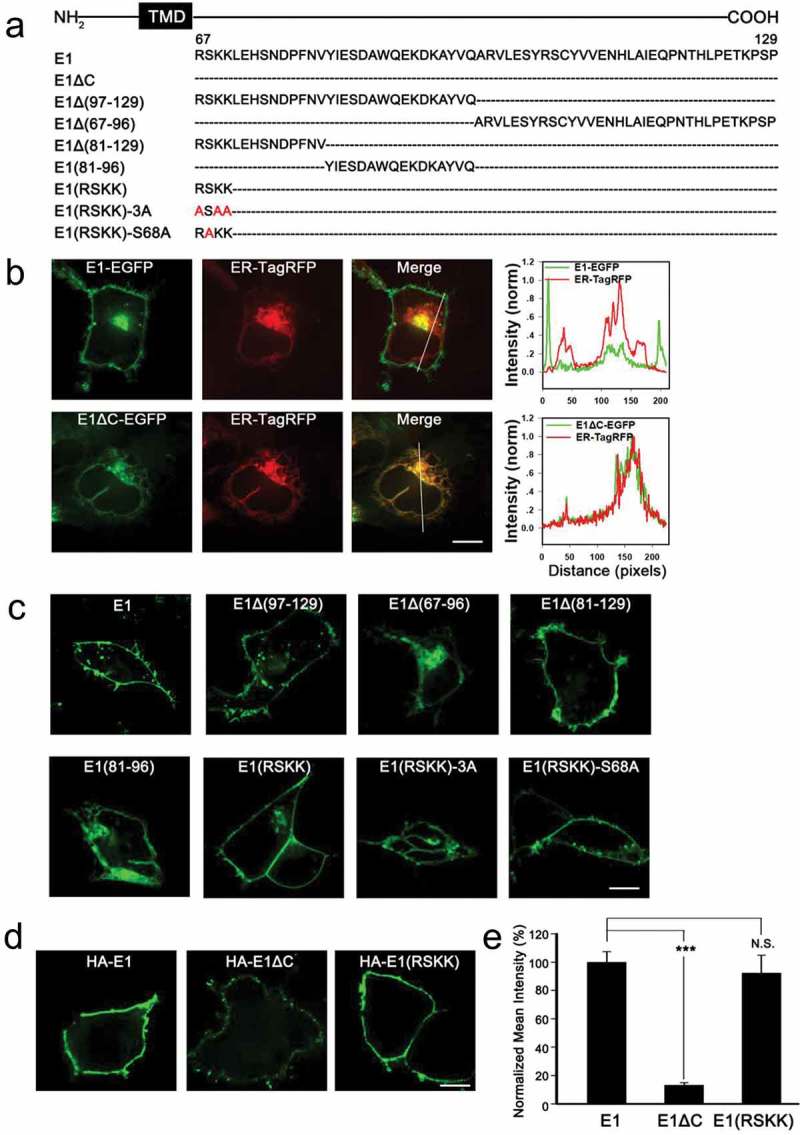
The conserved RSKK sequence in the C-terminus of KCNE1 is required for its efficient ER export. (a) C-terminal mutations of KCNE1 are displayed, where the dashed lines represent the deleted amino acids and the red represents the amino acids mutated to alanine. (b) Confocal images of HEK293 cells co-transfected with indicated E1* (WT and mutant E1)-EGFP and ER-TagRFP. The merged images show the combination. The scale bar is 10 μm. The right column shows the pixel intensity profiles of crossed sections indicated by the white line. (c) Subcellular localization of WT and mutant KCNE1 that were expressed as EGFP fusion proteins in HEK293 cells. The scale bar is 10 μm. (d) Immunofluorescence images of HEK293 cells transfected with HA-E1* as indicated. The cells were immunostained with mouse anti-HA primary antibody and goat anti mouse Alexa Fluor 488-conjugated second antibody under nonpermeabilized conditions. The scale bar is 10 μm. (e) Statistics of the relative surface fluorescence intensities of HA-E1* were plotted as indicated. n = 97, 86, 83 for E1, E1ΔC, E1(RSKK). *** p < 0.001, N.S. represents no significance (Student’s t-test).

KCNE1, another important member of the KCNE family, is also expressed clearly in the cell membrane in HEK293 cells, but the C-terminal (amino acid residues 67–129) deletion mutant E1ΔC showed intracellular retention and clearly colocalized with the ER marker (Figure 5b). Similar to KCNE2, the molecular basis for the subcellular segregation of KCNE1 was further investigated by examining a series of C-terminal truncations of KCNE1 (Figure 5a) that were fused with EGFP in HEK293 cells. As shown in Figure 5c, truncations containing only the RSKK sequence, E1Δ(97–129), E1Δ(81–129) and E1(RSKK), showed strong cell surface expression, while E1Δ(67–96) and E1(81–96) were largely retained in the cytoplasm. We also inserted a HA-epitope at the extracellular N-terminus of WT KCNE1 and its mutants (Figure 1a) to quantitatively measure their surface fluorescence intensities. The surface fluorescence intensity of HA-E1(RSKK) (92.3 ± 12.6%) was similar to that of HA-E1 (100.0 ± 7.4%), while the surface fluorescence intensity of HA-E1ΔC was reduced to 13% compared to that of HA-E1 (Figure 5d,e). These results suggested that the RSKK sequence is of great importance in regulating ER export of KCNE1 to the plasma membrane.

In addition, mutation of the residue S68 did not significantly alter the membrane targeting of E1(RSKK) (Figure 5c). However, when the three positively charged residues were mutated to alanine (Figure 5a), E1(RSKK)-3A displayed dramatic intracellular retention, indicating that the positively charged residues in RSKK were engaged in controlling the ER export of KCNE1 (Figure 5c).

### Role of the RSKK sequence in determining the function of KCNQ1/KCNE1 complex

**Figure 6.**
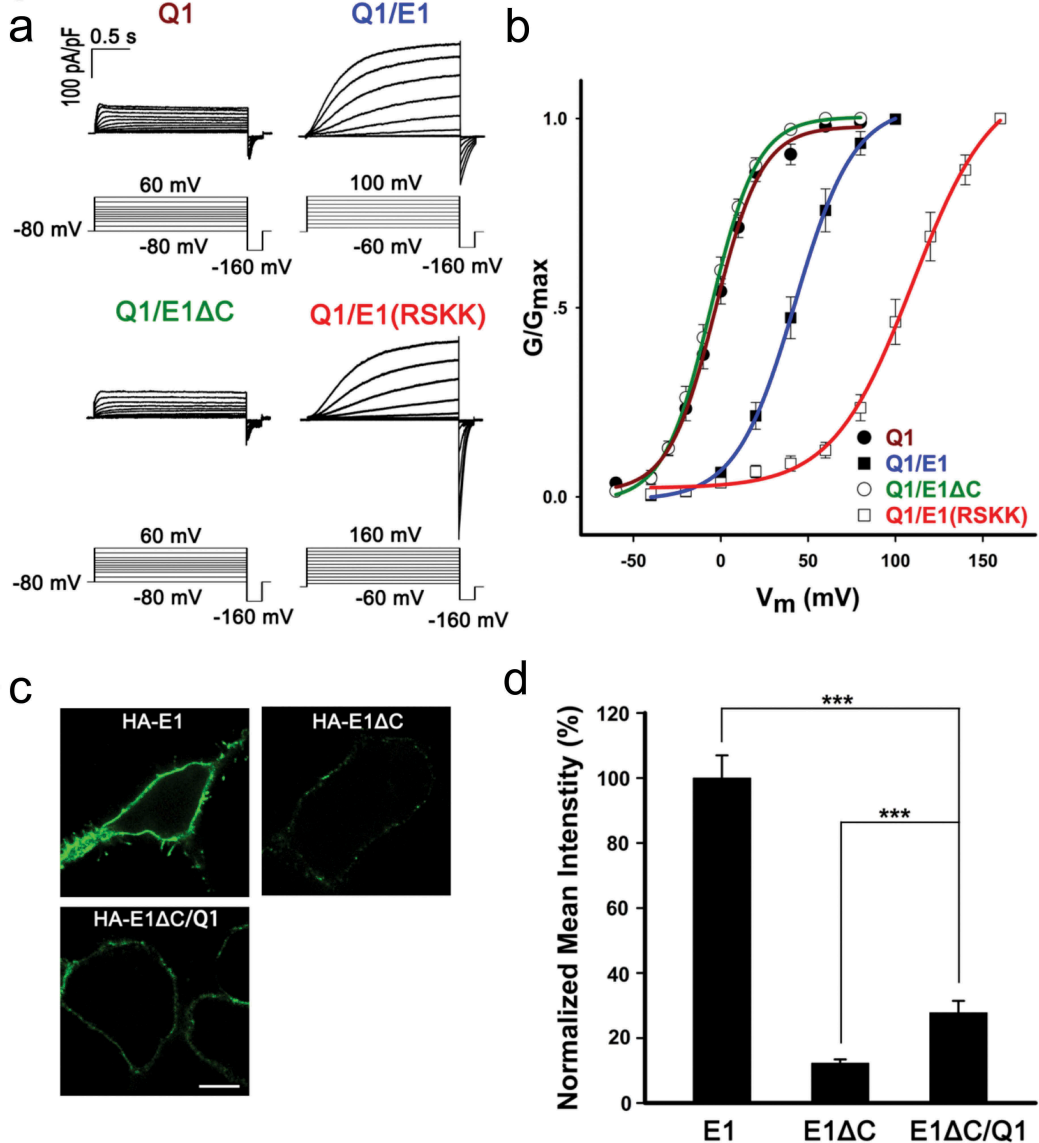
Role of the conserved sequence (RSKK) in determining the function of Q1/E1 complex. (a) Current traces of Q1 and Q1/E1* expressed in HEK293 cells. The voltage protocol was placed at the bottom. All the currents are shown as current density in pA/pF. (b) G-V curves of Q1 and Q1/E1*. Solid lines were fitted by single Boltzmann function. n = 7, 7, 6, 6 for Q1, Q1/E1, Q1/E1ΔC, Q1/E1(RSKK). (c) Immunofluorescence images of HEK293 cells transfected with HA-E1, HA-E1ΔC or HA-E1ΔC/Q1. The cells were detected with mouse anti-HA antibody and goat anti mouse Alexa Fluor 488-conjugated IgG under nonpermeabilized conditions. The scale bar is 10 μm. (d) Normalized fluorescence intensities of HA-E1* for the transfections of HA-E1, HA-E1ΔC or HA-E1ΔC/Q1. n = 149, 137, 141 for E1, E1ΔC, E1ΔC/Q1. *** p < 0.001 (Student’s t-test).

It has been reported that mistrafficking of KCNE1 could alter *I*_Ks_ modulation [31]. Therefore, we investigated the role of KCNE1 forward trafficking in regulating KCNQ1 channels. Electrophysiological studies were conducted in HEK293 cells, which were transfected with Q1, Q1/E1, Q1/E1ΔC or Q1/E1(RSKK), respectively. The cells transfected with Q1/E1 exhibited the well-known *I*_Ks_ currents characterized by slow activation kinetics, an increase in current density and a shift in voltage dependence of activation compared to Q1 alone (Figure 6a,b). Q1/E1(RSKK) also yielded slow-activated currents; compared to Q1/E1, the current density of Q1/E1(RSKK) decreased and the activation G-V curve of the channels shifted to more positive potentials by about 60 mV. In contrast, the current characteristics of Q1/E1ΔC were almost the same as Q1 (Figure 6a,b). These data indicated that the RSKK sequence is important for channel modulation, probably by affecting the plasma-membrane localization or gating modulation of KCNQ1/KCNE1 complex.

The surface expression of KCNE1 is increased when co-expressed with KCNQ1 in HEK293 cells [45]. Thus we questioned whether E1ΔC can form complexes with KCNQ1 to target the cell surface. We performed immunofluorescence experiments to examine the surface expression of E1ΔC in the presence or absence of KCNQ1. As shown in Figure 6c,d, a significantly higher intensity of cell surface staining of E1ΔC was observed in Q1/E1ΔC compared to E1ΔC alone, indicating that co-expression with KCNQ1 promotes E1ΔC trafficking to the cell surface. However, although a small amount of E1ΔC can associate with KCNQ1 and travel to the cell membrane, no *I*_Ks_ current was generated, whereby indicating that the RSKK sequence may play additional roles in modulating *I*_Ks_ except for regulating the ER export of KCNE1.

## Discussion

It is well established that the targeting and location of channels are important to determine their functions. In this study, we explored the roles of KCNE1 and KCNE2 C-terminus in regulating their subcellular localization, and demonstrated that the ER export of KCNE1 or KCNE2 depends on sequence information within its cytoplasmic C-terminus in HEK293 cells.

KCNE2 and KCNE1 presented membrane expression in HEK293 cells. But the C-terminal deletion mutants E2ΔC and E1ΔC were strongly retained in the ER (Figure 1b,5b). We identified a R/K-based motif, [R/K](S)[R/K][R/K], which is essential for the ER export of KCNE1 and KCNE2. This motif is highly conserved among all the five KCNE subunits. It has been reported that the dibasic motif, [R/K](X)[R/K], functions as an ER export signal by interacting with the Sar1 in many glycosyltransferases [23,27,28,43]. A recent study has also shown that a similar motif, RNKR, in *Drosophila* Crumbs contributes to the ER export by interacting with the Sar1 [46]. The conserved motif identified in our study is similar to these motifs. Therefore, we speculate that KCNE2 and KCNE1 are very likely to interact directly with the Sar1 through the [R/K](S)[R/K][R/K] motif presented at the C-terminus for ER export. It has also been suggested that the positively charged amino acids may associate with phospholipid components, which are present on the cellular membrane and involved in vesicle formation and trafficking [47], thus modulating ER export or plasma-membrane localization of membrane proteins [42,48]. Since the residues R67, K69, K70, and H73 in KCNE1 have been reported to be a key structural determinant of *I*_Ks_ phosphatidylinositol 4,5-bisphosphate (PIP2) sensitivity [49], so it cannot be ruled out that the positively charged amino acids in the proximal C-terminus promote the ER export of KCNE1 and KCNE2 through binding to PIP2 or other phosphoinositide species.

Unlike E2(KSKR) and E1(RSKK), E2ΔC and E1ΔC failed to regulate KCNQ1 channels to confer background currents or *I*_Ks_ currents (Figure 4, 6). This is not likely caused by a disruption of the physical interaction, because co-IP experiments demonstrated that the association between KCNQ1 and E2ΔC was not impaired compared to KCNQ1 and E2(KSKR); surprisingly, KCNQ1 and E2ΔC even co-precipitated to a higher degree (Figure S2). Similarly, the association of KCNQ1 with E1ΔC was greater than KCNQ1 with E1(RSKK) (Figure S3). We assumed that ER retention of KCNEΔC (E2ΔC or E1ΔC) increased the binding probability of KCNQ1 and KCNEΔC, analogous to previous study reported that addition of ER-retention/retrieval signal to the KCNEs led to larger association of the proteins with HERG [50], another E2 partner α-subunit channel. Besides, truncations of the C-terminus did not reduce the apparent affinity of KCNE2 for KCNQ1 (Figure S2), demonstrating our previous prediction that the rigid C-terminus of KCNE2 may not physically interact with the cytoplasmic regions of KCNQ1 [36]. In contrast, E1ΔC and E1(RSKK) showed a lower association for KCNQ1 channel (Figure S3), which is in accordance with previous finding that the KCNE1 C-terminus is required for the interaction with KCNQ1 [51,52].

ER retention of KCNEΔC resulting in greater association of proteins with KCNQ1 supports the concept that co-assembly of KCNE β-subunits with KCNQ1 channels probably begins in the ER [7,8,31,45]. It is suggested that the actual sites of final subunits co-assembly may also include Golgi [50]. Besides, vesicle injection experiments in oocytes indicated that free KCNE peptides delivered to plasma membrane are able to form functional channel complexes with KCNQ1 in the cell membrane [8,11]. Therefore, KCNQ1 channels could co-assemble with KCNE at multiple sites, which should largely depend on the temporal and spatial relationship between KCNQ1 and KCNE [8], while protein processing and trafficking of KCNE would have significant effects on association with KCNQ1 for channel modulation [31]. In our study, when KCNE2 (or E2(KSKR)) targeted the plasma membrane, the surface expression of KCNQ1 and KCNE2 (or E2(KSKR)) were both greatly decreased (Figure 4d,f), suggesting that KCNE2 strongly suppress KCNQ1 currents probably by inhibiting forward trafficking (ER retention or Golgi retention) or increasing internalization [53]. However, when E2ΔC was retained in the ER, although its association with KCNQ1 was even greater, the surface expression of KCNQ1 was not significantly impaired (Figure 4d), indicating that the process inhibiting surface expression of KCNQ1 by KCNE2 does not happen in the ER. Since the transport of KCNE2 to the cell surface should be rapid under normal conditions [50] and free KCNE2 peptides injected into KCNQ1-expressing oocytes can associate with KCNQ1 channels in the cell membrane to modulate their activities [8], the functional KCNQ1/KCNE2 complex is more likely formed in the cell membrane. As to the reduction in the surface expression of KCNQ1 and KCNE2, it is presumably because they are internalized after co-assembly in the cell membrane. Analogously, KCNE2 has been reported to reduce hERG surface expression by modulating hERG internalization [54]. Thus, the membrane targeting of KCNE2 is necessary for the binding of KCNQ1 and KCNE2 in the cell membrane to reduce KCNQ1 surface expression. This is parallel to the previous hypothesis that most KCNE2 should traffic to the cell surface with short transit times in the cytosolic compartments; the availability of KCNE2 subunits in the cell membrane is essential for the dynamic control of *I*_Ks_ current amplitude [8].

Unlike KCNE2, the surface expression of KCNE1 was enhanced when co-expressed with KCNQ1 [45]. Consistent with this, KCNQ1 co-expression increased E1ΔC membrane expression level even when E1ΔC was strongly sequestered in the ER (Figure 6d). This provides further evidence that co-assembly of KCNQ1 and KCNE1 may occur in the ER and then the channel complex traffics to the cell membrane. Nevertheless, co-transfection of KCNQ1 and E1ΔC did not produce slow-activated *I*_Ks_ current, where the current density and G-V curve were similar to that of KCNQ1 alone (Figure 6a,b). This is most probably because KCNE1 C-terminus is essential for modulating the gating properties of KCNQ1/KCNE1 complex as our previous study showed that E1[E2(C)], a KCNE1 chimera which was replaced the KCNE1 C-terminus with KCNE2 C-terminus, fully eliminated the *I*_Ks_ current when co-expressed with KCNQ1 [36]. However, co-transfection of KCNQ1 and E1(RSKK) can produce slow-activated current, indicating that the sequence RSKK play a crucial role in gating modulation of *I*_Ks._ It has been reported by other group that KCNE1 mutations (R67C, R67H, K70M, and K70N), which are associated with LQTS, could reduce *I*_Ks_ current [49]. We postulate that the decrease of *I*_Ks_ current caused by these mutations maybe partially due to the mistrafficking of KCNE1, supported by a study showing that an ER-targeted KCNE1 (KCNE1-ER) reduced *I*_Ks_ current about 80% compared to WT KCNE1/KCNQ1 [31].

In summary, we identified a conserved R/K-based motif, [R/K](S)[R/K][R/K], in the juxtamembranous C-terminal region that positively regulates the ER export of KCNE1 and KCNE2 in HEK293 cells. The results indicated that ER export of KCNE1 and KCNE2 is vital for regulating KCNQ1 function. Additionally, the KCNE1 C-terminus is crucial for its interaction with KCNQ1, whereas the KCNE2 C-terminus is not. Our study provides significant insight into the trafficking determinants of KCNE β-subunits and how they bind and modulate KCNQ1 channels.
