# Supplementary material for: A conserved arginine/lysine-based motif promotes ER export of KCNE1 and KCNE2 to regulate KCNQ1 channel activity
Source: Channels (Austin). 2019 Nov 3;13(1):483–97. doi: 10.1080/19336950.2019.1685626 (PMC6833972; doi:10.1080/19336950.2019.1685626)
Supplement: Supplemental Material [file kchl-13-01-1685626-s001.docx]

**A conserved arginine/lysine-based motif promotes ER export of KCNE1 and KCNE2 to regulate KCNQ1 channel activity**

Bin Hu^1,#^, Wen-Ping Zeng^1,2,#,※^, Xia Li^1,#^, Umar Al-Sheikh^1^, San-you Chen^1,3^, Jiu-Ping Ding^1,※^

^1^Key Laboratory of Molecular Biophysics of the Ministry of Education, College of Life Science and Technology, Huazhong University of Science and Technology, Wuhan, Hubei, 430074, China

^2^School of Life Sciences, University of Science and Technology of China, Hefei, Anhui, 230027, China

^3^CAS Key Laboratory of Microscale Magnetic Resonance and Department of Modern Physics, University of Science and Technology of China, Hefei, Anhui, 230026, China

^#^The authors contributed equally to this work.

^※^To whom correspondence may be addressed: jpding@hust.edu.cn or zengwenping@126.com.

**Figure S1**


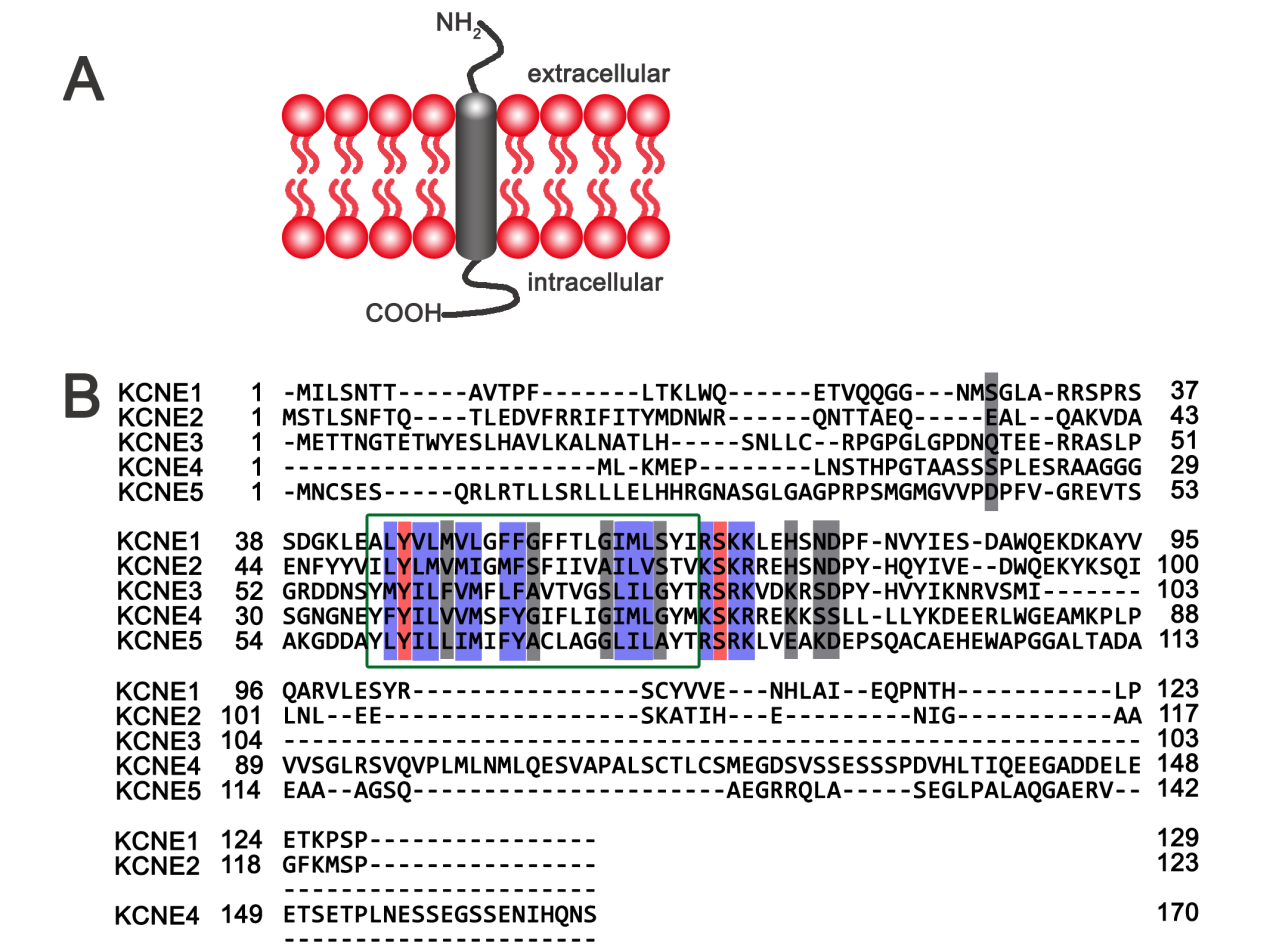


**Figure S1. KCNE β-subunits. (A)** Topology diagram of KCNE β-subunits. **(B)** Amino acid sequence alignment of human KCNE family (http://www.uniprot.org/align). Transmembrane domain (TMD) was displayed in the green box. The degree of conservation observed in each column is marked with different colors (red, fully conserved residue; blue, strongly conserved residues; gray, weakly conserved residues).

**Figure S2**


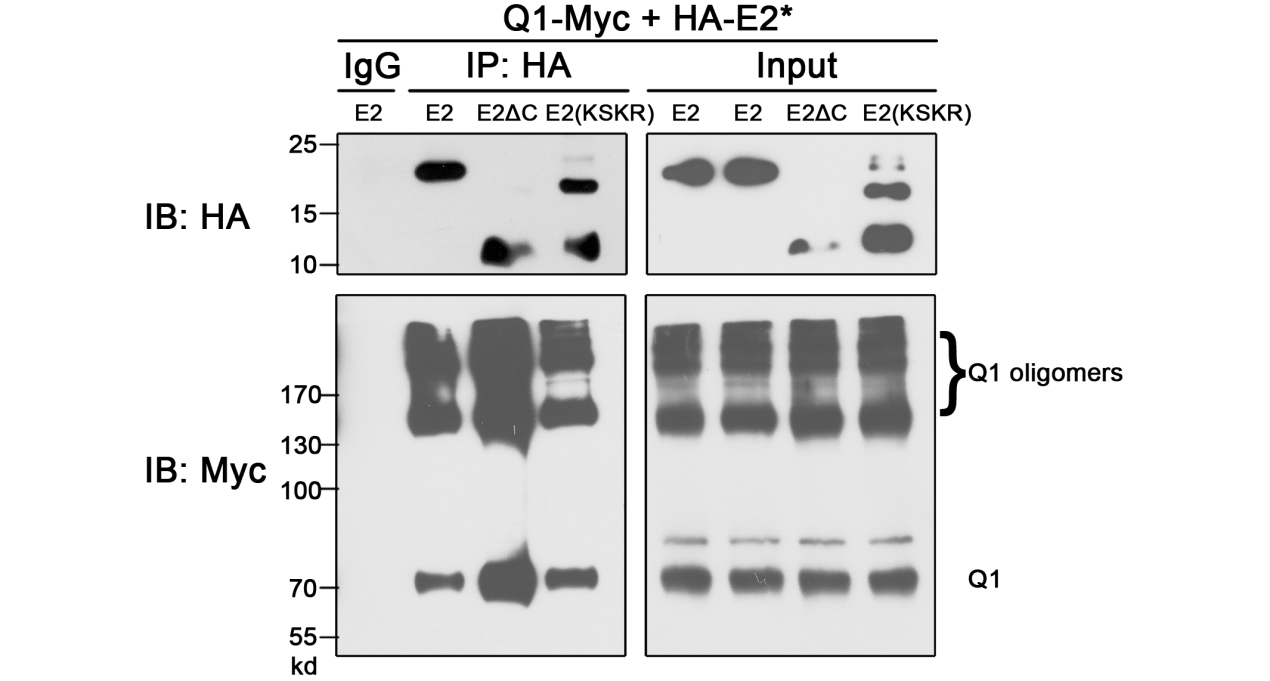


**Figure S2. Co-immunoprecipitation of HA-E2* with Q1-Myc.** Q1-Myc was expressed with HA-E2*. HA-E2* was immunoprecipitated with mouse anti-HA antibody (IP: HA) as indicated, and western blot analysis was performed with HRP-conjugated mouse anti-HA antibody. Q1-Myc was detected with rabbit anti-Myc antibody. The lane marked “input” refers to the whole cell lysate from co-expressed HEK293 cells. The lane “IgG” represents a negative control sample, which was immunoprecipitated with mouse normal IgG.

**Figure S3**


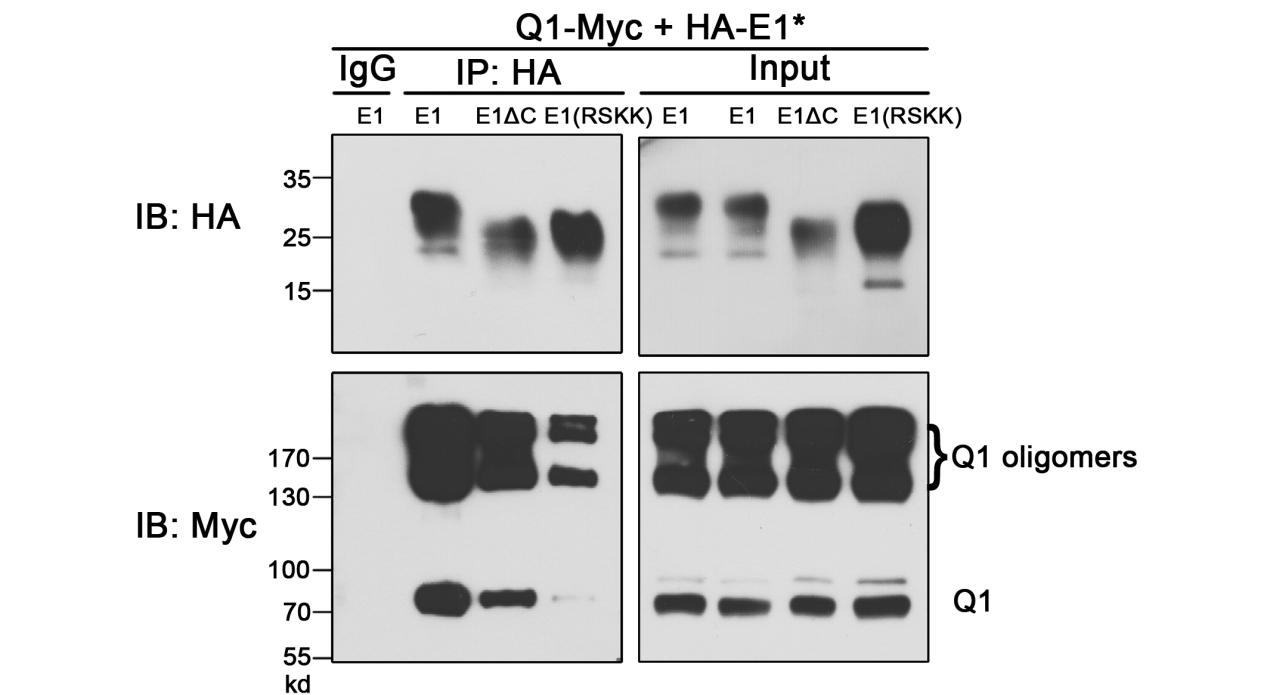


**Figure S3. Co-immunoprecipitation of HA-E1* with Q1-Myc.** Q1-Myc was expressed with HA-E1*. HA-E1* was immunoprecipitated with mouse anti-HA antibody (IP: HA), and western blot analysis was performed with HRP-conjugated mouse anti-HA antibody. Q1-Myc was detected with rabbit anti-Myc antibody. The lane marked “input” refers to the whole cell lysate from co-expressed HEK293 cells. The lane “IgG” represents a negative control sample, which was immunoprecipitated with mouse normal IgG.
